# Supplementary material for: Non‐canonical metabolic pathways in the malaria parasite detected by isotope‐tracing metabolomics
Source: Mol Syst Biol. 2021 Apr 6;17(4):e10023. doi: 10.15252/msb.202010023 (PMC8022201; doi:10.15252/msb.202010023)
Supplement: Supplementary file 1 — Appendix [file MSB-17-e10023-s008.pdf]

Multiplex isotope-tracing metabolomics of Plasmodium falciparum asexual blood stages reveals extensive metabolite damage-repair systems

Simon A Cobbold[1], Madel V Tutor[1*], Philip Frasse[3*], Emma McHugh[1], Markus Karnthaler[1], Darren J Creek[4], Audrey Odom John[2], Leann Tilley[1], Stuart A Ralph[1], Malcolm J McConville[1]

1Department of Biochemistry and Molecular Biology, Bio21 Institute of Molecular Science and Biotechnology, University of Melbourne, Parkville, Victoria, Australia
2 The Children's Hospital of Philadelphia and University of Pennsylvania, Philadelphia, PA, USA
3Department of Medicine, Washington University School of Medicine, St. Louis MO USA
4Monash Institute of Pharmaceutical Sciences, Monash University, Parkville, Victoria, Australia
*Authors contributed equally to the work

Appendix Table of Contents -

Appendix Figure S1. The percent label and pool size of glycolytic intermediates

Appendix Figure S2. 13C network reconstructions for lysine and arginine

Appendix Figure S3. 13C network reconstructions for amino acid mix

Appendix Figure S4. 13C network reconstructions for bicarbonate

Appendix Figure S5. 13C network reconstructions for acetate

Appendix Figure S6. 13C network reconstructions for glycerol

Appendix Figure S7. PCR confirmation of integration

Appendix Figure S8. 2,3,3-D-Serine and 13C-glycine labelling into P. falciparum-infected RBCs.

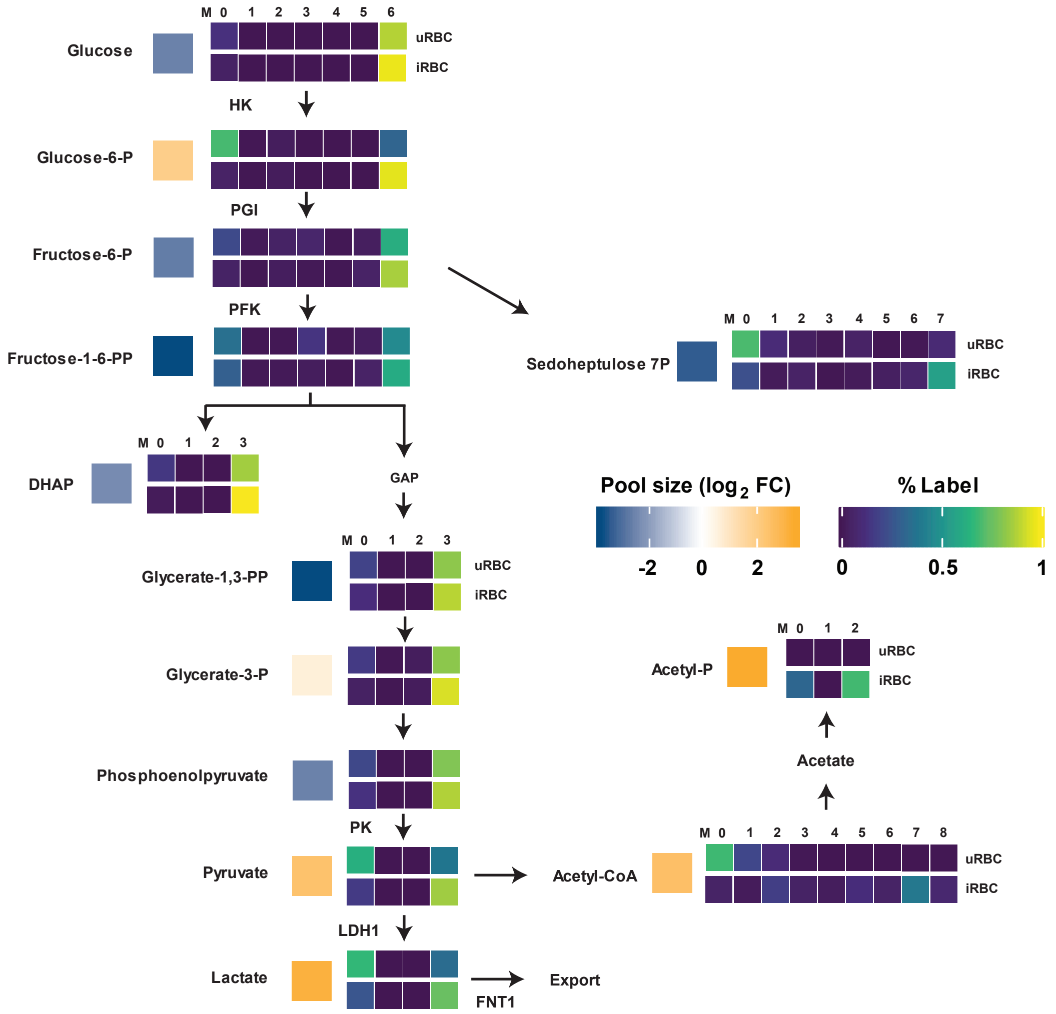

Appendix Figure S1. The percent label and pool size of glycolytic intermediates. Relative metabolite pool size is depicted as the log2 fold change (iRBC/uRBC) and the percent enrichment of 13C-incorporation for each isotopologue are presented.

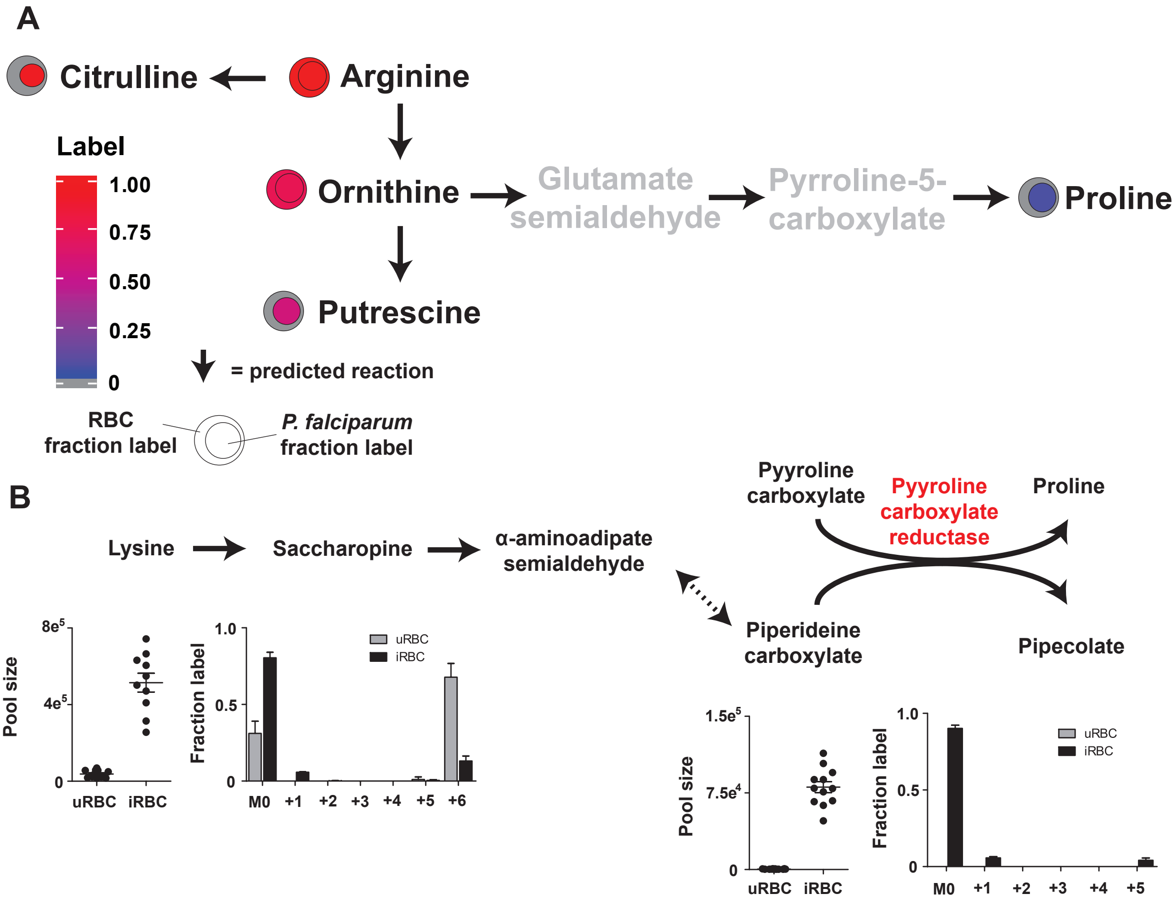

Appendix Figure S2. 13C network reconstructions for lysine and arginine. A) 13C-arginine labelling. B) 13C-lysine labelling

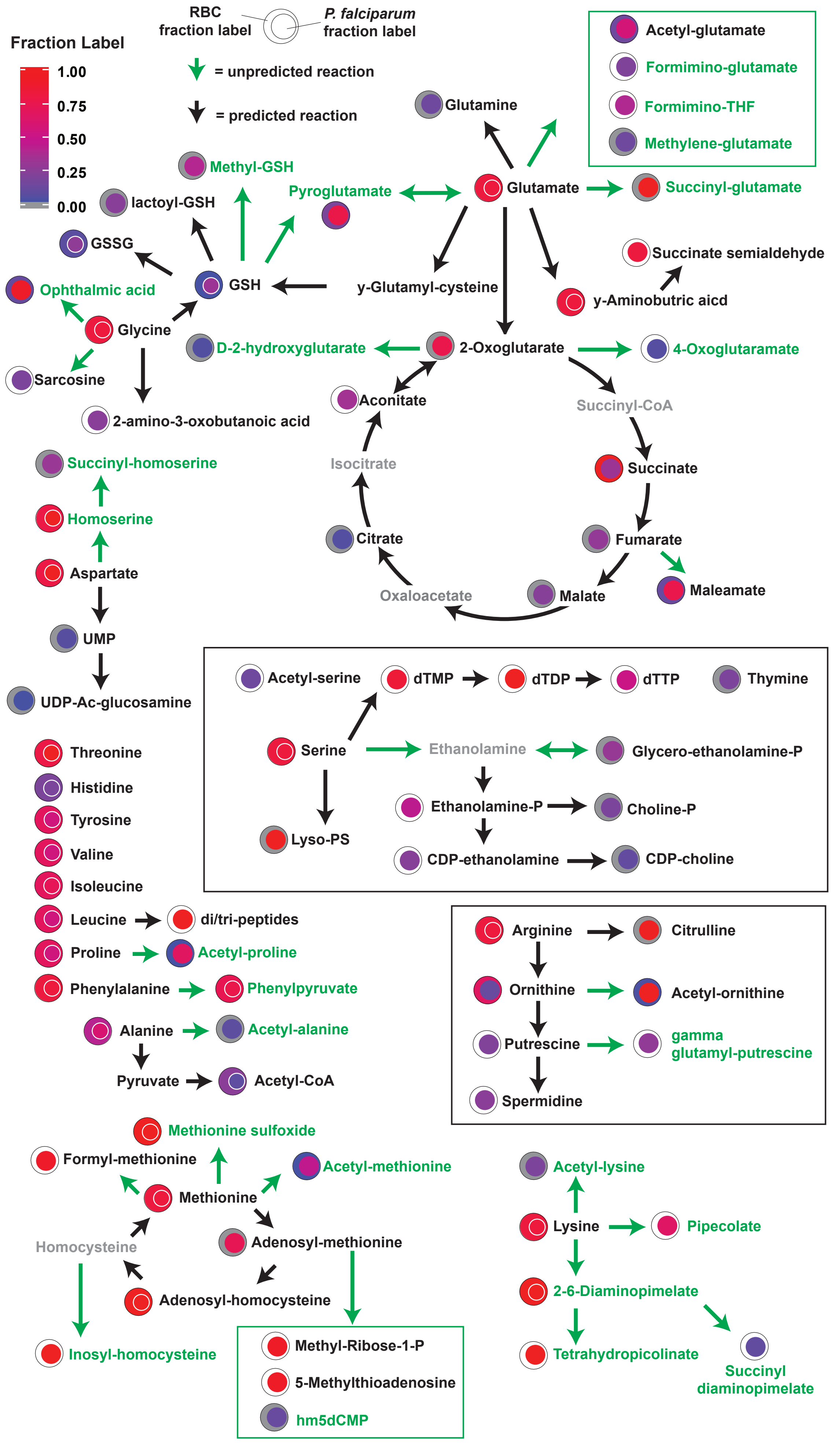

Appendix Figure S3. 13C network reconstructions for amino acid mix

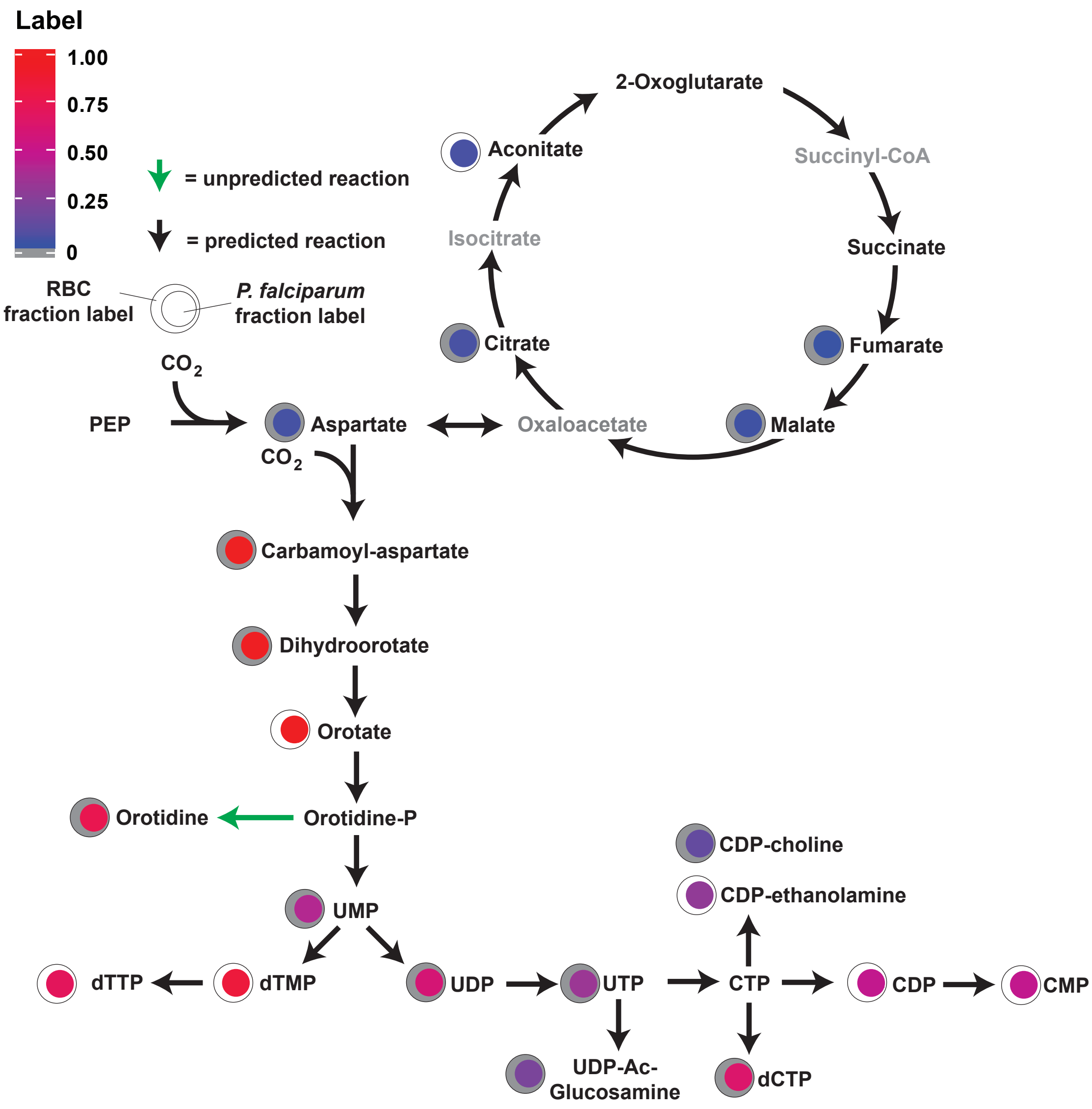

Appendix Figure S4. 13C network reconstructions for bicarbonate

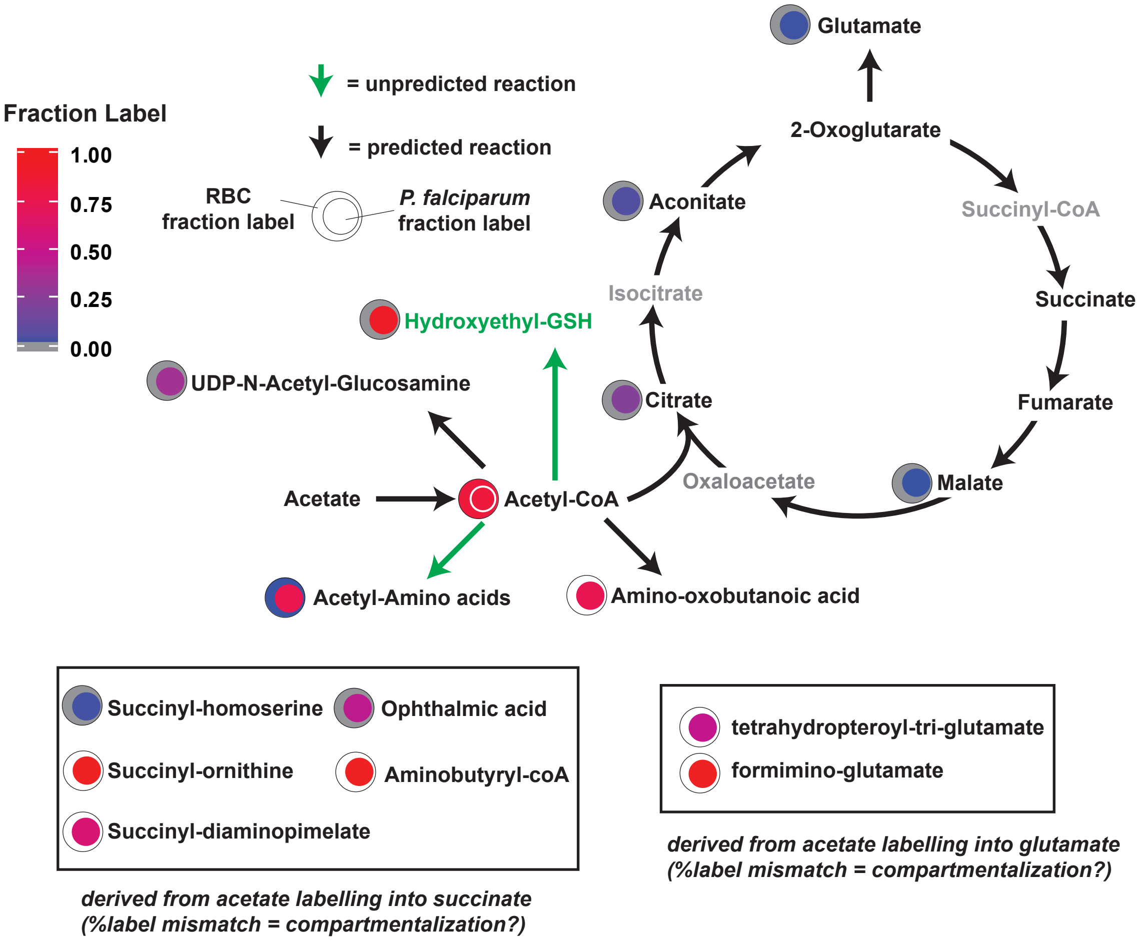

Appendix Figure S5. 13C network reconstructions for acetate

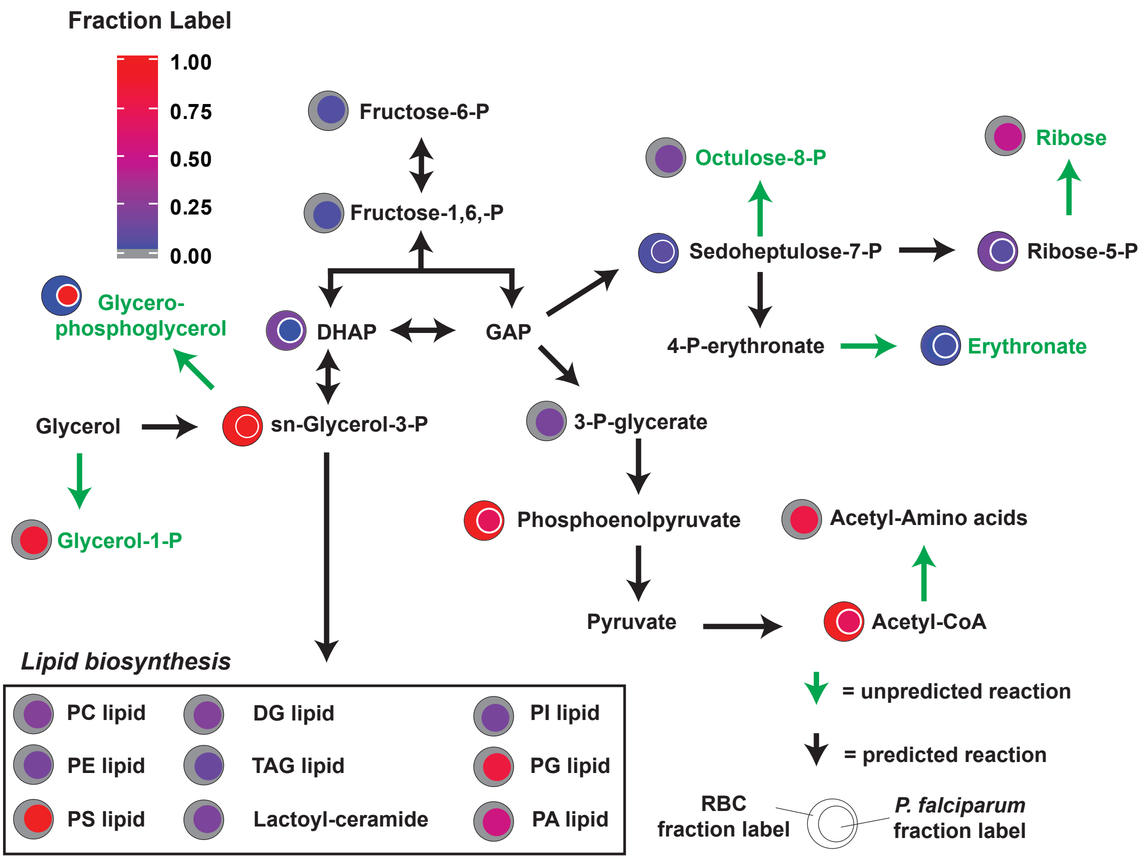

Appendix Figure S6. 13C network reconstructions for glycerol

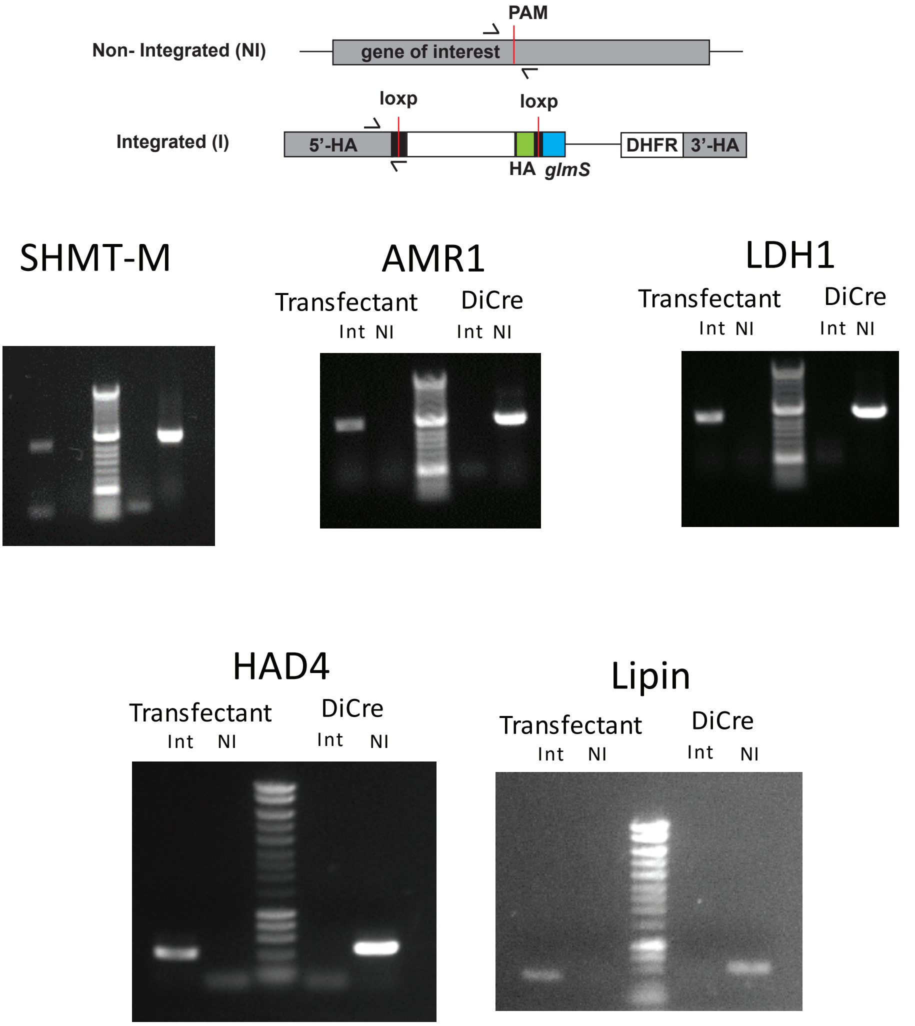

Appendix Figure S7. PCR confirmation of integration. 'Int' indicates primers specific for the integrated locus and 'NI' indicates primers specific for the endogenous (non-integrated) locus. DiCre represents the untransfected parental line stably expressing the DiCre recombinase.

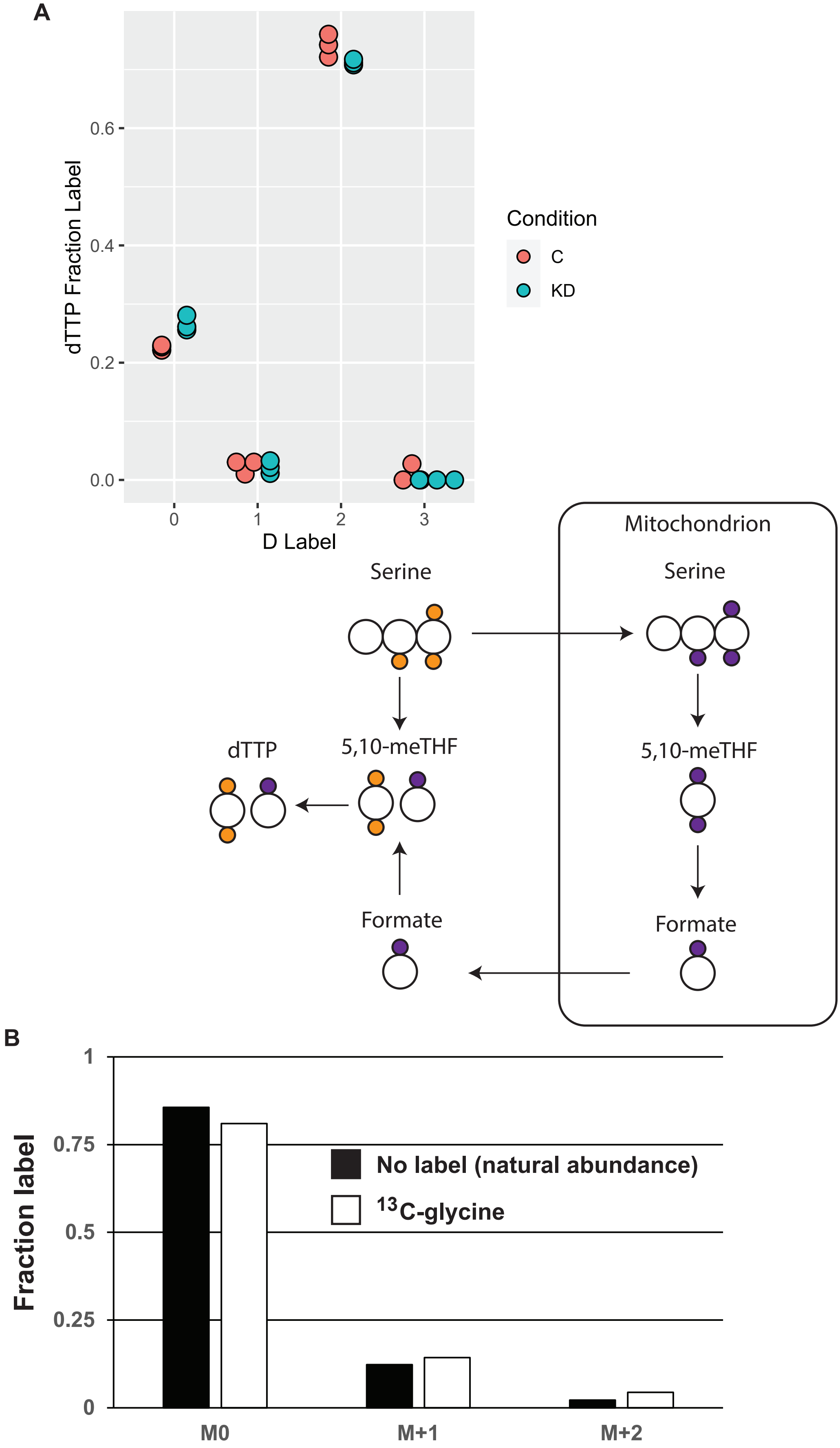

Appendix Figure S8. A. 2,3,3-D-Serine labelling into *P. falciparum*-infected RBCs. Cleavage of 2,3,3-D-serine via SHMT yields a one-carbon unit bound to tetrahydrofolate (5,10-methylene-THF) with two labelled protons. Subsequent oxidation within the mitochondrion yields formate with one labelled proton. Export of mitochondrial formate into the cytosol and metabolism into dTTP yields a signature labelling pattern for the cytoplasmic (M+2) and mitochondrial (M+1) origin. **B.** 13C-glycine labelling into P. falciparum-infected RBCs. dTTP labelling was monitored as a recipient of any one-carbon production via the glycine-cleavage system in the mitochondrion.
