# Supplementary material for: Non‐canonical metabolic pathways in the malaria parasite detected by isotope‐tracing metabolomics
Source: Mol Syst Biol. 2021 Apr 6;17(4):e10023. doi: 10.15252/msb.202010023 (PMC8022201; doi:10.15252/msb.202010023)
Supplement: Supplementary file 2 — Expanded View Figures PDF [file MSB-17-e10023-s006.pdf]

Expanded View Figures

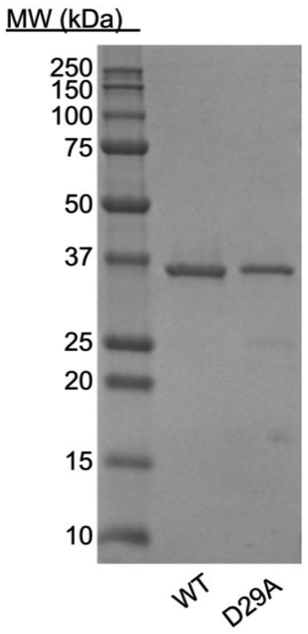

Figure EV1. Coomassie-stained SDS–PAGE gel of purified HAD4.
Source data are available online for this figure.
