# Supplementary figures and images for: Non‐canonical metabolic pathways in the malaria parasite detected by isotope‐tracing metabolomics
Source: Mol Syst Biol. 2021 Apr 6;17(4):e10023. doi: 10.15252/msb.202010023 (PMC8022201; doi:10.15252/msb.202010023)

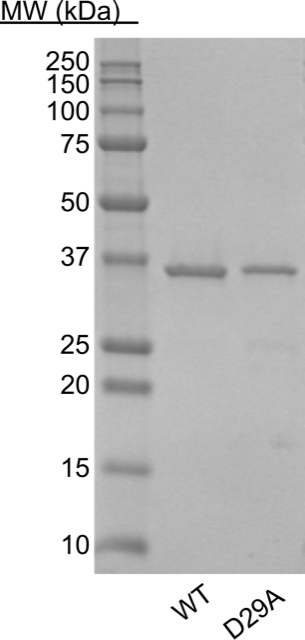

Supplement: Supplementary file 10 — Source Data for Appendix [file MSB-17-e10023-s009.zip › msb202010023-sup-0009-SDataFigEV/msb202010023-sup-0009-SDataFigEV1.png]
